# ADJUVANTED VACCINES TO ADDRESS IMMUNOSENESCENCE AND PROMOTE HEALTH THROUGHOUT LIFE

**DOI:** 10.1093/geroni/igz038.772

**Published:** 2019-11-08

**Authors:** Leonard Friedland

**Affiliations:** GSK Vaccines, Philadelphia, Pennsylvania, United States

## Abstract

Immunosenescence creates a challenge in developing vaccines tailored for older adults. Recent advances in immunology, molecular biology and systems vaccinology have enabled greater understanding of the innate and adaptive immune mechanisms behind vaccine responses in adults. Novel approaches to vaccine design for this population include adjuvant technology. New knowledge and accumulating experience enables evidence-based selection of the right antigen and adjuvant combination leading to the discovery and development of better and/or newer innovative vaccines specifically tailored to protect against pathogens responsible for infectious diseases resulting in significant morbidity and mortality in older adults. For example, the recent licensure of several vaccines formulated with a new generation of adjuvants to help protect older adults against influenza, hepatitis B and herpes zoster (shingles). This evidence-based approach to the development of adjuvanted vaccines addressing immunosenescence is a primary prevention strategy to develop and maintain the functional ability that enables wellbeing in older age.

